# When Can One Vaccinate with a Live Vaccine after Wild-Type Dengue Infection?

**DOI:** 10.3390/vaccines8020174

**Published:** 2020-04-09

**Authors:** Bruno Guy, Eng Eong Ooi, Jose Ramos-Castañeda, Stephen J. Thomas

**Affiliations:** 1 Consultant, 69000 Lyon, France; 2 Program in Emerging Infectious Diseases, Duke-NUS Medical School, Singapore 169857, Singapore; engeong.ooi@duke-nus.edu.sg; 3 Instituto Nacional de Ciencias Medicas y de la Nutrcion “Salvador Zubiran”, (INCMNSZ), Departamento de Infectologia, Ciudad de Mexico 14080, Mexico; jose.ramos@infecto.mx; 4 Departamento de Inmunidad, Instituto Nacional de Salud Publica, Cuernavaca, Morelos 62100, Mexico; 5 Institute for Global Health and Translational Sciences, Upstate Medical University, State University of New York, Syracuse, NY 13210, USA; thomstep@upstate.edu

**Keywords:** dengue, live vaccine, vaccination decision, multidose

## Abstract

Recommendations have been issued for vaccinating with the Sanofi Pasteur tetravalent dengue vaccine (CYD-TDV, Dengvaxia^®^) individuals aged from 9 to 45/60 years old with a prior dengue virus (DENV) infection and living in endemic countries/areas. One question linked to these recommendations is to determine when it is possible to start vaccination after laboratory confirmed wild-type DENV infection, and this question can be relevant to any live vaccine to be used in endemic areas. To address it, we reviewed and discussed the immunological and practical considerations of live vaccination in this context. Firstly, the nature and kinetics of immune responses triggered by primary or secondary DENV infection may positively or negatively impact subsequent live vaccine take and associated clinical benefit, depending on when vaccination is performed after infection. Secondly, regarding practical aspects, the “easiest” situation would correspond to a confirmed acute dengue fever, only requiring knowing when the patient should come back for vaccination. However, in most cases, it will not be possible to firmly establish the actual date of infection and vaccination may have to take place during well-defined periods, regardless of when prior infection occurred. Evidence that informs health authorities and medical practitioners in formulating vaccine policies and implementing vaccine programs is thus needed. The present work reviewed the different elements of the guidance and proposes some key conclusions and recommendations.

## 1. Introduction

The Sanofi Pasteur chimeric yellow fever-dengue tetravalent dengue vaccine (CYD-TDV, Dengvaxia^®^) is used for the prevention of acute dengue caused by any of the four serotypes of dengue virus (DENV). This vaccine is licensed for use in individuals aged from 9 to 45 or 60 years old and living in endemic countries/areas with confirmed prior DENV infection. This population with prior DENV infection is at risk of secondary DENV infection, which is associated with increased risk of severe and hospitalized dengue.

When vaccinating those with prior DENV infection, an important question that needs to be addressed is when after wild-type infection can a person be vaccinated? How early can it be performed? This question applies to any live vaccine to be used in endemic areas.

To address this question, one first needs to present the nature and kinetics of immune responses triggered by dengue wild type infection, primary or secondary, and how such responses may impact, positively or negatively, subsequent live vaccine take and associated clinical benefit, depending on when vaccination is performed after infection.

The present document will not address or discuss the ways prior DENV infection (symptomatic or not) would have been assessed and determined. One will take as a starting point that prior infection did take place and was laboratory confirmed, thus leading to the decision to vaccinate. In this context, one could encounter different situations. The “easiest” one would correspond to a confirmed acute dengue fever: when can vaccination be performed after such an acute infection? When should the patient come back? However, it is likely that the actual date of infection cannot firmly be established in most instances. In this regard, the present document aims at guiding individual practitioners in making a decision on when to vaccinate. Our findings would, we believe, also be of interest to a broader audience, including health authorities establishing vaccine policies at the country level. These aspects will be discussed in Section 4.

## 2. Kinetics of Immune Responses Induced after Wild Type Dengue Infection

### 2.1. Primary Infection

Primary infection with DENV induces potent innate immune responses, which will exert direct antiviral activities and also shape humoral (antibody production by B cells) and cellular (mediated by CD4+ and CD8+ T cells) responses that can be either DENV serotype-specific or cross-reactive against different serotypes (for reviews see [1,2,3]).

#### 2.1.1. Innate Responses

Innate responses represent the first line of defense and particularly involve monocytes, macrophages and myeloid dendritic cells, the latter being critical antigen presenting cells (APCs) in the initiation of primary responses. Infection also impacts additional arms of the innate immune system, triggering the expression of a wide array of pro-inflammatory (or anti-inflammatory) cytokines and chemokines, which level and kinetics will shape the outcome of the disease and of subsequent adaptive responses (for reviews see [4,5,6]). Innate responses reach a peak in the early febrile phase and then rapidly decline; however, some factors/cytokines can remain elevated during defervescence and convalescence stages, up to four weeks after infection [7].

#### 2.1.2. Adaptive Responses

##### Antibody Responses

Upon primary infection, IgM antibodies appear first, between day 3–10 of illness; IgM levels peak about 2 weeks after the onset of fever and then generally decline to undetectable levels over the next 2–6 months. Dengue-specific IgG responses can already be detected by day 7 of illness and then slowly increase. Antibodies can be both specific to the infecting serotype and cross-neutralizing. It appears, however, that cross-reactive antibodies may decline faster than serotype-specific ones; it was observed that after primary infection children developed serotype–cross-neutralizing antibody responses that narrowed to a single serotype over a 12-month period [8]. The breadth of neutralization then progressively declined following infection, in agreement with other studies showing that cross-neutralizing antibodies become progressively monotypic with time [9,10]. This may impact subsequent vaccine take, as discussed below.

##### Cellular Responses

While the kinetics of DENV-specific CD4+ and CD8+ T cell responses are not as well characterized as anti-DENV antibodies, such responses triggered in parallel to B cell responses can play a beneficial role in recovery, depending on their specificity and profile. The main targets of anti-dengue CD8+ cells are nonstructural (NS) proteins, while both CD4+ and CD8+ responses can be directed against structural proteins and also play a role in protection/recovery (for reviews see [1,2,6,11]). Regarding CD8+ cells in particular, their ability to sterilize dengue infection has recently been observed in a lymphopenic renal transplant recipient [12]. Therefore, both B and T cell memory responses, serotype-specific and cross-reactive, will play an important role upon subsequent infection (or vaccination).

**Figure 1:**
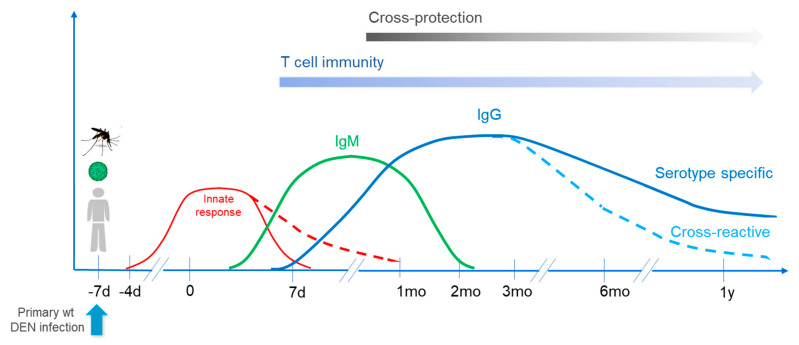
Upon primary wild type dengue infection, innate responses develop early and peak during the first week following onset of fever, while some responses can persist for several weeks. Dengue specific IgM responses develop first, followed by IgG. IgM responses usually wane after two months and IgG responses persist a longer time, while cross-reactive responses wane more rapidly. CD4+ and CD8+ T cell responses are also triggered during the early phase of infection, and T/B cell memory is then established. Induced responses support cross-protection (see below), which can last up to 3 years (more generally 6 months to 2 years). d: day; mo: month; y: year; wt: wild type.

The kinetic of responses following primary wild type infection is presented in Figure 1.

### 2.2. Secondary Infection

Antibody responses induced by secondary infections (homologous or heterologous) present differences in kinetics, magnitude, quality, and specificity as compared to those after primary infection. IgG responses are induced faster and at higher levels following a secondary compared to primary infection; responses rise dramatically over an approximately 2-week period after secondary dengue. On the other hand, IgM response are usually lower in secondary infections [2]. Regarding antibody specificity, secondary responses induced by heterologous serotypes present a high proportion of cross-neutralizing antibodies targeting conserved epitopes different from those targeted after primary infection, resulting in higher affinity responses [9,10,13,14,15,16].

### 2.3. Cross-Protection

#### 2.3.1. Natural Situation in the Field

Immunity induced by primary infection is believed to induce life-long protection against the infecting serotype (homotypic protection); a small number of homotypic re-infections have been documented [17,18]. On the other hand, cross-protection (heterotypic protection) following a primary infection may last from several months to up to 3 years (more generally from 6 months to 2 years), corresponding to a transient protective “honeymoon” or “grace” period [19,20,21,22], which in the present document will be referred to as a “refractory period” regarding subsequent vaccination. This period is followed by a period of higher susceptibility to severe disease; as mentioned above, as cross-reactive responses decline over time along with the potential for cross-protection.

Protection after secondary infection also presents some differences compared to protective response following primary infection. Following secondary infection levels of induced antibodies are higher, with a broader specificity and higher affinity. Their cross-protective activity can persist for a longer period of time after secondary infection, with sustained T and B cell memory. There is consensus in the field that post-secondary infections (i.e., third or fourth infections) are mostly asymptomatic (for a review see [23]).

**Figure 2:**
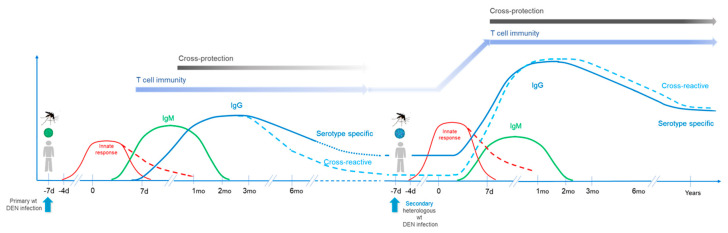
Upon secondary heterologous wild type dengue infection, innate responses develop early as after primary infection, but may present higher levels and a more inflammatory profile. Dengue specific IgM responses still develop, possibly at lower levels than after primary infection. IgG responses are triggered/boosted earlier than upon primary infection and reach higher levels. Of importance, cross-reactive responses raise to higher levels too, also presenting some higher affinity and different specificity. CD4+ and CD8+ T cell responses are also triggered/boosted, and as for IgG responses present a broader specificity. These responses then support longer and more efficient cross-protection. d: day; mo: month; y: year; wt: wild type.

These aspects are summarized in Figure 2.

#### 2.3.2. Experimental Human Infection (Challenge) Studies

While human challenge studies do not fully recapitulate the human infection experience which occurs in the field, they can nevertheless be informative. In the case of dengue, experimental human infections have been in the literature since 1902 and renewed interest for such an approach has triggered more recent developments (see [24]).

Sabin and colleagues completed a series of experiments during the World War II era [25], and their laboratory notebooks were subsequently systematically reviewed by Snow et al. [26]. With respect to the present question—how long a prior infection can block a subsequent live infection/vaccination—Sabin’s work reanalyzed by Snow et al. brings relevant information. Subjects primarily infected with either DENV-1 or DENV-2 were then secondarily ‘cross-infected’ at various timepoints with either DENV-2 or DENV-1 respectively, and their signs and symptoms of dengue disease were followed. The data showed self-limiting fever in subjects who received their secondary infection 4 months or later after the primary infection, while secondary challenge performed within shorter time intervals did not trigger clinical infection, except for one case with moderate fever. Significant limitations exist however in Sabin’s study. In particular, the lack of tools to titrate the viral inoculum and measure the antibody response to infection—the size of the inoculum may indeed impact in one way or another the magnitude of innate responses and subsequent adaptive ones. The method of virus delivery (i.e., mosquito versus needle) could also impact infection outcome. Nevertheless, Sabin’s data, as observed for natural infections in the field, further show that a “refractory period”, lasting at least several months, follows DENV infection.

#### 2.3.3. Sterilizing Immunity vs. Protection against Symptomatic Disease

When talking about a refractory period, one has also to consider that it may correspond to a true sterilizing immunity—preventing infection/replication and presentation of viral antigens to adaptive immune cells, and then not boosting previous immunity—or to protect against symptomatic disease, without totally preventing infection. In this latter case, some replication and viremia may occur, then boosting immunity to some extent.

Therefore, sterilizing immunity would totally prevent vaccine take while partial blockade may nevertheless allow the establishment of a certain level of vaccine-induced immunity. This may depend on the time interval between infection and vaccination—and the persistence of some innate and adaptive B and T cell responses—and would also be impacted by the nature of the prior wild type infecting serotype: sterilizing immunity may more likely occur against the homotypic serotype present in the vaccine, while leaving more room to the other serotypes to replicate.

These points will be discussed in the following section.

## 3. What Is the Impact of Dengue-Induced Pre-Existing Responses on Subsequent Vaccine Safety and Clinical Benefit? How Long Should One Wait after Wild Type Infection before Vaccinating?

### 3.1. Safety

Before addressing clinical benefit, one needs to ask whether pre-existing immune responses induced by primary or subsequent wild type infections impact vaccine reactogenicity/safety.

First, regarding innate responses, it has been observed that the Sanofi Pasteur vaccine did trigger antiviral responses in innate dendritic cells, but not inflammatory cytokines as compared to wild-type viruses, which induced a predominantly inflammatory profile [27]. Therefore, whichever the time the vaccine is administered after infection, it would not increase the inflammatory responses induced by previous or intercurrent wild type infection.

Regarding overall reactogenicity in the field, pooled safety data were analyzed from 18 phase I, II and III clinical trials in which the dengue vaccine was administered to participants aged 2–60 years, including long-term safety follow-up in three efficacy trials. It was shown that baseline dengue virus serostatus did not influence the safety profile of the vaccine nor vaccine viremia [28]. While, as will be discussed below, a potential explanation to the higher vaccine immunogenicity observed in pre-immune subjects could be linked to cross-reactive antibodies enhancing vaccine take [29], there was nonetheless no increased rates of adverse events.

Collectively, these findings show that no safety impact from prior infection is expected after vaccination, whichever the time interval considered.

### 3.2. Clinical Benefit

#### 3.2.1. Negative Impact of Prior Infection

A “refractory period” is expected to follow wild type infection, preventing or lowering vaccine take and associated clinical benefit, and involves different arms of immunity.

##### Impact of Innate Responses

As mentioned above, innate antiviral responses are present during the first days following infection and can still be detected after several weeks. They can thus impact negatively—and potentially totally block—the replicative vaccine take. Therefore, regardless of adaptive responses, a strong early innate response, if induced, would be able to fully prevent infection and further presentation of vaccine antigens to adaptive cells.

One could expect that vaccinating before one month after infection would be mostly inefficient.

##### Impact of Adaptive Responses

- Impact of antibody responses/Primary infection

Considering live vaccines, one can anticipate that pre-existing and significant levels of serotype-specific or cross-reactive antibody responses may block their infectivity and subsequent replication, and therefore limit adaptive immune response to the vaccine. One should then wait for a certain period of time after infection to ensure a proper induction of vaccine-induced immunity. Some studies and trials can be mentioned in this regard:

⚬ Monkey studies performed with the Sanofi Pasteur vaccine candidate have shown that in this species immune responses could benefit from a second vaccine dose administered two months after a live priming performed by a monovalent attenuated vaccine (“wild-type like” priming) or performed by a primary dose of a bivalent or tetravalent vaccine. Administering a second dose with a tetravalent or complementary bivalent vaccine two months after such monovalent, bivalent, or tetravalent priming was able to induce a strong booster effect with a broader response covering all four serotypes [30].
⚬ Some of these prime/boost combinations were then further tested in clinical trials. In humans, a second complementary bivalent dose administered 3.5 months after the first bivalent one was, however, largely inefficient and apparently blocked by the pre-existing cross-neutralizing immune response induced upon the first administration [31]. Therefore, the “refractory” period appears to be longer in humans as compared to monkeys, and this was also pointed out by other authors, who proposed to apply a longer interval between two live vaccine administrations, i.e., 6 months or longer [32]. Such a time interval should then also be applied to a first live vaccine administration following a natural infection.

This proposed 6-month time interval between infection and vaccination (refractory period) is also supported by field studies (“grace period” of cross-protection), as well as by Sabin’s human challenge studies (see above). Therefore, while primary vaccine administration during the first month following infection could be totally inefficient because of the innate immune response, vaccinating within the first 6 months could also be of reduced value because of cross-neutralizing immune responses; vaccinating during the first three months would be rather inefficient, while vaccinating 4–6 months after infection should bring at least moderate benefit. Overall, while one cannot rule out a positive effect of early vaccine administration on B/T memory during this refractory period, it could nevertheless be more efficient to start vaccination from 6 months post-infection.

As mentioned above, one needs to distinguish between sterilizing immunity and immunity allowing some viral replication and then further immune stimulation: following wild type infection with a given serotype, sterilizing immunity may more likely occur against the homotypic serotype present in the vaccine, while leaving more room to the other serotypes to replicate and trigger immune responses. Such sterilizing immunity against the prior infecting serotype would however not be an issue with regards to protection, as homotypic protection afforded by wild type infection is proposed to be life-long in most cases.

In many cases, one will not know, however, when wild-type infection did take place (most infections are asymptomatic). Issues related to unknown timing of prior DENV infection will be discussed in Section 4.

- Impact of antibody responses/Secondary infection

When considering vaccination after secondary infection, one can propose that, due to higher and broader pre-existing immunity, the refractory period could be longer. However, as discussed in Section 4 below, the problem of the refractory period should be mitigated by a multidose vaccine which injections are performed several months apart (e.g., ≥ 6 months), ensuring that even if the first dose falls into that period, subsequent dose(s) would fall out of it. Therefore, even following secondary infections, such a vaccine would ensure induction of some significant immunity with a likely benefit on B and T memory, allowing to boost and maintain it. Supporting such a vaccine benefit after secondary infection, it was observed that the Sanofi Pasteur vaccine efficacy against virologically confirmed dengue and hospitalization was similarly high in volunteers presenting a monotypic or a multitypic profile based on measured baseline dengue PRNT_90_ assay; the former volunteers were considered to have experienced a single infection while the latter were considered to have experienced at least two infections [33]. 

Besides a higher titer, the epitopes targeted by cross-neutralizing antibodies that develop post-secondary infection appear also to be different compared to post-primary infection, as mentioned above [9,10,13,14,15,16], and this could also impact differently vaccine take. Further studies are needed to address this question. 

While it is still beneficial to perform vaccination in post-secondary dengue situations, the benefit afforded by vaccination will however be maximal after primary infection, preventing the potentially more severe dengue from secondary wild-type DENV infection (for a review see [23]). In any case, it will not be required when performing pre-vaccination serological screening to determine whether a positive anti-DENV IgG signal indicates prior primary or post-primary infections.

- Impact of T cell responses

While this paper focuses on the impact of wild-type induced antibody responses, one needs to also consider the impact of dengue-specific CD4+ and CD8+ responses induced in parallel. Such responses do impact subsequent dengue infections (see above Section 2, and ref [11]) and may similarly modulate vaccine take and benefit. 

One specificity of CYD-TDV is that it possesses a yellow fever 17D (YF17D) backbone, and thus expresses NS antigens of yellow fever virus. Accordingly, while CYD-TDV is able to induce cellular responses against dengue structural antigens and then provides benefit against dengue infection, potent CD8+ responses are induced against the YF17D NS3 protein, with only limited cross-reactivity with Dengue NS3, at least upon immunization in naïve volunteers [34,35]. Therefore, when considering the potential negative impact of dengue-specific cellular responses on CYD-TDV, one could propose that this impact would be more limited, because of the heterologous nature of the YF17D NS antigens, which are the dominant targets of CD8+ responses.

#### 3.2.2. Positive Impact of Prior Dengue Virus (DENV) Infection

We have so far considered the negative impact on vaccine benefit of prior wild type dengue infection, but one needs to also consider that such pre-immunity can also enhance vaccine-induced immunity.

Supporting this, a Sanofi Pasteur integrated immunogenicity analysis across 10 phase II and 6 phase III trials undertaken in dengue endemic and non-endemic countries confirmed that Geometric Mean Titers (GMTs) were positively influenced by baseline dengue seropositivity: participants who were seropositive to at least one dengue serotype at baseline in general achieved higher post-dose 3 GMTs for all serotypes than those who were seronegative regardless of the region [36].

This is can be due to a “booster” effect linked to previous immunity and memory (for a review see [23]). Different mechanisms could be linked to such a booster effect, such as induction of cross-reactive CD4+ cells supporting cross-reactive B cell responses, as seen between different flaviviruses [37], and/or through a boosting effect linked to cross-reactive antibodies allowing a better vaccine take and infectivity. This has been seen for instance in subjects presenting a specific range of cross-reactive antibody titers from a prior inactivated Japanese encephalitis (JE) vaccination and then developing enhanced Yellow Fever (YF) immunogenicity upon YF17D vaccination [29]. Regarding this latter mechanism, one has to consider however that only a limited range of cross-reactive antibody titers boost dengue or YF17D vaccine infection: at either end of the cross-reactive antibody spectrum, reduced vaccine infection and immunogenicity was observed [29,38,39].

In addition, or alternatively, CYD-TDV vaccination could act mechanistically as a secondary infection and the post-vaccination adaptive immunity profile would thus be akin to post-secondary infection subjects.

While prior infection history will have to be documented through viral diagnostic or serological assays such as Rapid Diagnostic Tests before vaccination, it is of course not intended to assess the level and specificity of individual neutralizing responses through quantitative assays such as PRNT_50_. One can however, based on clinical evidence across a large number of studies [36], propose that after the refractory period, pre-existing responses would in majority enhance vaccine immunogenicity.

**Figure 3:**
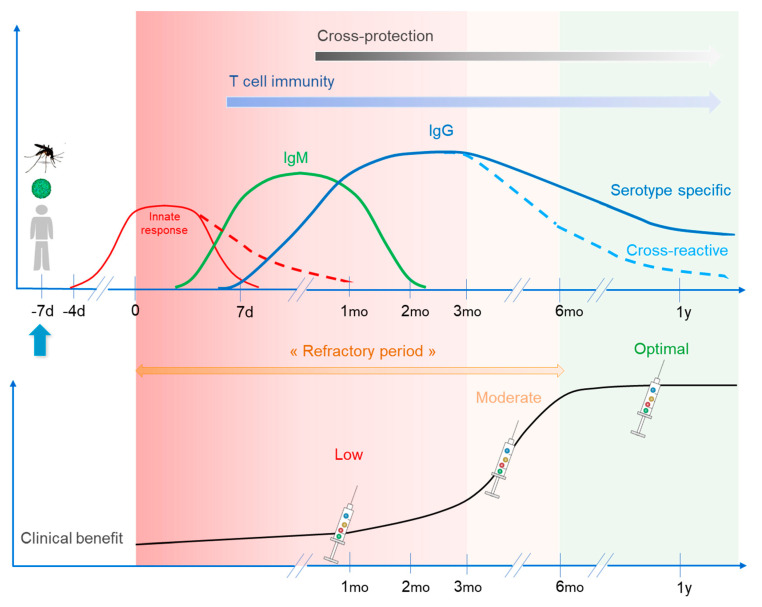
Wild type infection-induced responses will install a “refractory period” during which they would block or lower live-attenuated vaccine take and subsequent immunity/efficacy. Innate response would totally block any vaccine replication should it be given during the initial steps of infection, but IgM/IgG and T cell responses could still hamper vaccine take during the following weeks and months. When wild type-induced responses wane—cross-reactive responses in first place—this leaves more room for live vaccines to establish proper immunity, and they can even enhance it.

Figure 3 illustrates and summarizes the points developed above, addressing the introduction of a multidose vaccine in presence of infection-induced immune responses, and Table 1 summarizes the nature and potential role/impact of innate and adaptive responses induced by wild type dengue infection regarding subsequent live vaccination.

### 3.3. Impact of Prior Vaccination against Other Flaviviruses

The present document addresses the impact on vaccine benefit of prior dengue infection, and not of prior vaccination against other pathogens. One can nevertheless briefly mention some results regarding prior vaccination against other flaviviruses close to dengue viruses, such as Yellow fever (YF) or Japanese encephalitis (JE) viruses.

Information in this regard is more limited, but one trial showed no negative impact of prior immunity induced within a short interval of time by the YF17D vaccine on a Japanese encephalitis chimeric vaccine (JE-CV, using as CYD-TDV a YF17D backbone), and vice versa [40]. Different combinations were tested: YF17D followed by JE-CV 30 days later, JE-CV followed by YF17D 30 days later, or co-administration of JE-CV and YF17D, and none of these combinations significantly impacted in one or the other way induced responses. Similarly, co-administration of YF17D and CYD-TDV did not result in negative interferences at the serological level [41,42]. A larger study looked at the impact of prior YF or JE immunity on CYD dengue-induced antibody responses [36]. This study was conducted across ten phase II and six phase III trials undertaken with the CYD dengue vaccine in endemic and non-endemic countries. After ruling out all dengue seropositive participants, the number of vaccines who were dengue seronegative but seropositive for either JE or YF at baseline was limited. Consequently, a potential increase in dengue immune response because of prior exposure to these flaviviruses could not be clearly established nor ruled out. Information was also lacking in most cases regarding time intervals between the two vaccinations.

Cellular immunity was also looked at in a recent study, which evaluated the impact of prior or concomitant administration of inactivated JE vaccine on the immune responses induced by the CYD dengue vaccine administered on different schedules. It was observed that giving JE vaccine concurrently with the CYD dengue vaccine did not result in an increase in overall neutralizing antibody titers, while the increase in activated CD8+ T cells observed after CYD dengue vaccination was the greatest for the JE vaccine primed individuals [43].

Overall, at this stage, additional studies would be needed to better address the interplay between such vaccinations performed within different time windows.

## 4. Practical Aspects: Vaccination in the Field

When can one vaccinate with a live vaccine after a wild type dengue infection? 

The above sections addressed in a large part that question in the context of well-defined and controlled clinical studies and laboratory investigations. The situation is different in the field where one needs to apply the simplest and most practical rules regarding this type of vaccine.

We took as a starting point that prior dengue infection would have been laboratory-confirmed, whichever the way/tool used to get such confirmation. The above sections suggest that the time interval between wild-type dengue infection and vaccination can have a significant impact, but such an interval will be difficult to firmly establish in real life. As mentioned in Introduction, the “easiest” situation would correspond to a confirmed acute dengue infection, but most infections are asymptomatic, and, even when dengue infection could be suspected to have occurred at one given time, symptoms can be caused by other pathogens (e.g., malaria, flu, chikungunya, or more recently Zika): it is therefore likely that in the absence of confirmation at the time of infection, the actual date of dengue infection will be unknown in many cases.

There are however numerous situations where vaccination should be performed for practical reasons even in the absence of any information regarding the actual date of dengue infection. Even when the date is known with certitude, it could be necessary still for practical reasons, to start vaccination before the end of the 6-month refractory period.

In this regard, situations could be different according to who is performing vaccination and where: private versus public sector/program, doctor’s office, hospital, or school. Each of these scenarios would pose a different amount of room for flexibility. For instance, vaccination could be performed at least one month before the start of the rainy season in a given country/region, i.e., before dengue cases rise, thus during periods well-defined in advance, with little latitude to perform vaccinations outside these periods.

One can also discuss the possibility to vaccinate seropositive individuals at the very beginning of an outbreak, in order to protect them before any contact with the wild-type DENV. This could be beneficial, as protective responses would be triggered rapidly in primed individuals. However, if by chance vaccination would be performed during incubation or shortly after infection, this would mostly be inefficient as discussed in the above sections. Nonetheless, given the small likelihood of such coincidence, it should be emphasized that the negative impact on population level vaccine efficacy should remain limited. Nevertheless, while rapidly performing vaccination in such an outbreak situation could be possible, we suggest that vaccinating before the start of rainy seasons would be a more pragmatic approach to reduce the magnitude of epidemics.

This being said, vaccinating in well-defined periods could be problematic with respect to the refractory period discussed in the previous sections; however, in the case of a multidose vaccine in which doses are given 6 months apart, if the first dose falls within the refractory period, the second dose should fall outside that period. This type of regimen may then ensure that proper immunization and associated benefit will take place whenever the vaccination is done, thus allowing this type of vaccine to be used during such well-defined programs and periods.

Finally, another question could be raised: one could consider that waiting for 6 months post-infection would delay the possibility of benefitting from vaccine-induced immunity in endemic areas. However, as mentioned in Section 2, natural infection should provide at least a 6-month cross-protection against all four serotypes and then starting vaccination after this period should not be an issue. 

## 5. Conclusions

The present document presents a short summary of wild type dengue virus-induced responses and kinetics, and how they can interfere with live vaccine-induced immunity when vaccination is performed at different times after infection. We propose the following conclusions:

- Innate and adaptive immune responses triggered by dengue wild type infection should have no impact on vaccine reactogenicity/safety, whichever the time vaccination is performed after infection
- Innate and adaptive immune responses triggered by dengue wild type infection may induce a “refractory period” during which vaccination efficacy could be limited or even absent
  ⚬ To be fully efficient, primary vaccination should then not take place before 1 month post-infection, and preferably not before 6 months
  ⚬ Performing vaccination after 3 months could nevertheless provide some benefit
- If the actual date of infection is unknown, or if for practical reasons vaccination would be performed before this 6 month period of time (i.e., 3–5 months), this would not be an issue in the case of multidose vaccine schedules, in which doses are given 6 months apart, as the second dose would induce a proper and efficient immune response outside of the refractory period
- In this regard, vaccinating before the start of rainy seasons would be a pragmatic approach to reduce the magnitude of epidemics
- Waiting for 6 months to start vaccination after wild-type infection would also not be an issue regarding induction of protection, as infection induces at least a 6 month to 1-year cross-protection

## Figures and Tables

**Table 1:** Synthetic view of the different types of responses induced after wild-type dengue infection, and of their potential impact on subsequent live vaccination. Duration of such responses is only indicative and depend on multiple host and viral parameters. Antigen and epitope specificity may also depend on the considered serotype for both B and T cell responses. The level and nature of responses also depend on the primary or post-primary nature of viral infection. *: existence of B and T cell memory.

Type of Dengue-Induced Responses			Role	Duration (Average)	Potential Impact on Vaccination	Refs
**Innate**			• Antiviral response • Inflammation • Shapes subsequent adaptive responses	• 1—4 weeks	• Block vaccine take and subsequent responses	[1,2,3,4,5,6,7]
**Adaptive**	Antibodies	Serotype-specific	• Prevent serotype-specific infection • If too low levels, may enhance infection	• Years *	• Block vaccine take of the corresponding serotype • Among a certain range, may favor vaccine responses • Memory responses may be boosted by the vaccine	[1,2,3,8,9,10]
		Cross-reactive	• Prevent cross-serotype infection • If too low levels, may enhance infection (also depends on epitope specificity and affinity)	• 6 months to 1–2 years *	• Block/lower cross-serotype vaccine take • Among a certain range, may favor vaccine responses • Memory responses may be boosted by the vaccine	[1,2,3,8,9,10,13,14,15,16]
	Cellular	CD4	• Help antibody and CD8 responses against structural and nonstructural antigens • Antiviral responses through cytokines and possibly cytotoxicity • May be deleterious, depending on cytokine profile (if too inflammatory or immunosuppressive; depends also on epitope specificity and affinity)	• Months to years *	• Block vaccine take through direct cytokine (cytotoxic) activity and/or through rapid help to B and CD8 cells • Depending on level and specificity, may favor vaccine-induced responses (helper activity) • Memory responses may be boosted by the vaccine	[1,2,6,11,12]
		CD8	• Eliminate infected cells (cytotoxicity) • Block viral dissemination • Improve recovery • Target in majority nonstructural antigens • May be deleterious, depending on cytokine profile (if too inflammatory or immunosuppressive; depends also on epitope specificity and affinity)	• Months to years *	• Block vaccine take through destruction of infected cells and/or direct cytokine activity • Memory responses may be boosted by the vaccine	[1,2,6,11,12]
